# Molecular validation and redescription of *Glypthelmins staffordi* (Digenea: Plagiorchiidae) with an updated key to genus *Glypthelmins* Stafford, 1905

**DOI:** 10.1007/s00436-026-08668-4

**Published:** 2026-04-10

**Authors:** Abigail Hui En Chan, Urusa Thaenkham, Chanisara Kaenkaew, Vachirapong Charoennitiwat, Wallop Pakdee

**Affiliations:** Laboratory of Helminth Biodiversity and Drug Development, Department of Helminthology, Faculty of Tropical Medicine, Mahidol University, Bangkok, Thailand

**Keywords:** *Glypthelmins*, Molecular identification, Morphology, Taxonomy

## Abstract

*Glypthelmins* is a genus comprising species that parasitize anurans and has been reported in America and Asia. The taxonomic history of the genus *Glypthelmins* is problematic, involving the reclassification of species into other genera. *Glypthelmins staffordi* was consequently not considered a valid species in *Glypthelmins* due to morphological differences and was placed in the genus *Rauschiella*. Thus, the valid name of *G. staffordi* prior to this work is *R. staffordi*. Moreover, no molecular data has been published for *R. staffordi*. To address this gap, a morphological redescription of *G. staffordi* is provided, along with the confirmation of the phylogenetic placement of *G. staffordi* in genus *Glypthelmins* using three genetic markers. Morphological comparisons were conducted with eight other *Glypthelmins* species, and the molecular phylogeny revealed that the *G. staffordi* specimens form a monophyletic group with other *Glypthelmins. Glypthelmins staffordi* was also evidenced to be genetically distant from genus *Rauschiella*. Thus, *G. staffordi* is proposed to be reinstated as a valid species within the genus *Glypthelmins*, instead of in *Rauschiella*. Additionally, the morphological character of the excretory vesicle should not be used as a key character to distinguish among genera. Supplementing the systematics of this taxonomically complex group, we provide an updated key for the genus *Glypthelmins*, alongside molecular sequences of *G. staffordi*, to supplement the identification of *Glypthelmins* species.

**Supplementary Information:**

The online version contains supplementary material available at 10.1007/s00436-026-08668-4.

## Introduction

*Glypthelmins* Stafford 1905; a genus within the family Plagiorchiidae, contains species parasitizing the gastrointestinal tract and liver of anurans (Razo-Mendivil and Pérez-Ponce de León 2008). Species of *Glypthelmins* are morphologically characterized as having tegument armed with scale-like spines, I-shaped excretory vesicle, having an oral sucker which is larger in size than the ventral sucker, where the latter is positioned in the anterior third of the body (Bray et al. 2002; Razo-Mendivil and Pérez-Ponce de León 2008; Schell 1970).

Currently, *Glypthelmins* contain eleven species, with *Glypthelmins quieta* Stafford 1905 as the type species (Razo-Mendivil and Pérez-Ponce de León 2008; Stafford 1905). The eleven species are: *G. quieta*, *G. californiensis* (Cort 1919), *G. parva* Travassos 1924; G. *intestinalis* (Lucker 1931), *G. shastai* Ingles 1936; G. *facioi* Brenes, Madrigal, Arroyo Sancho, Jimenez-Wuiroz and Delgado Flores, 1959, *G. pennsylvaniensis* Cheng 1961; G. *hyloreus* Martin 1969; G. *brownorumae* Razo-Mendivil, Leon-Regagnon and Pérez-Ponce de León, 2004, *G. tuxtlasensis* Razo-Mendivil, Leon-Regagnon and Pérez-Ponce de León, 2004, and *G. eleutherodactyli* Moravec and Prouza 2024.

The taxonomic history of *Glypthelmins* has been unstable, with several species originally classified in this genus later transferred to other genera including *Margeana* Cort 1919, *Haplometrana* Lucker 1931; and *Rauschiella* Babero, 1951 (Babero 1951a, b; Cheng 1959). Cheng (1959) proposed to separate the species based on the absence or presence of peripharyngeal glands, transferring those without peripharyngeal glands to *Margeana*. As a result, only three species remain in *Glypthelmins*, where *Glypthelmins staffordi* Tubangui 1928; was placed in *Margeana* due to the absence of peripharyngeal glands (Cheng 1959; Tubangui 1928). Subsequently, *G. staffordi* was proposed to be in *Rauschiella*, due to its Y-shaped excretory vesicle (Babero 1951a). Consequently, *R. staffordi* was not included in *Glypthelmins*, with the latest redescription of *G. staffordi* done by Yuen (1962) and no further morphological descriptions or molecular information regarding this species have been provided thus far (Yuen 1962). Thus, prior to our research, *G. staffordi* is in *Rauschiella*, known as *R. staffordi*.

*Rauschiella staffordi* was previously reported in *Rana vittigera* Wiegmann 1834 in the Philippines and China, *Occidozyga lima* Gravenhorst 1829 in Vietnam, *Rana cancrivora* Gravenhorst 1829, *Rana erythaea* Schlegel 1837 and *Rana macrodon* Duméril and Bibron 1841 in Singapore and Malaysia, *Bufo melanostictus* Schneider 1799 in China, and *Hoplobatrachus rugulosus* Wiegmann 1834 in Vietnam, China, and Thailand (Cheng 1959; Fischthal and Kuntz 1967; Li 1937a, b; Miquel et al. 2018; Qiu 2019; Sullivan 1976; Syogaki 1937; Yamaguti and Mitunaga 1943; Yuen 1962). In Thailand, *R. staffordi* was previously recorded infecting *H. rugulosus* from Udon Thani (Miquel et al. 2018). However, majority of them were reported many years ago without supporting molecular information. Here, through molecular validation using nuclear and mitochondrial genetic markers, we confirm the phylogenetic placement of *R. staffordi* in genus *Glypthelmins* instead of *Rauschiella and* provide a morphological redescription of the specimens obtained from *H. rugulosus* in Thailand. An updated key to genus *Glypthelmins* is also provided to supplement the systematics of this taxonomically challenging group.

## Methods

### Parasite isolation

A total of three *Hoplobatrachus rugulosus* were purchased from a local market in Min Buri District, Bangkok, Thailand. The frogs, which were not alive prior to purchase, were dissected to examine parasites present in the mesentery and intestine. The adult trematodes were isolated and counted, and a subset was selected for morphological analysis (preserved in 10% near-boiling formalin), and another subset was kept in 70% ethanol for molecular analyses.

### Morphological analysis

Fifteen adult trematodes were stained in acetic carmine and subsequently mounted in permount slides for morphological examination. The characters and morphometrical measurements were examined using an inverted compound microscope equipped with a digital camera and image analysis software (ZEISS Primovert, Germany). The specimens were then compared with *G. staffordi* and eight other *Glypthelmins* species (*G. intestinalis*, *G. californiensis*, *G. facioi*, *G. brownorumae*, *G tuxtlasensis*, *G. eleutherodactyli*, *G. parva*, and *G. shastai*) (Cheng 1959; Fischthal and Kuntz 1967; Ingles 1936; Lucker 1931; Moravec and Prouza 2024; Razo-Mendivil et al. 2004; Sullivan 1976; Yuen 1962). These eight species were selected from the eleven currently recognized *Glypthelmins* species as they lack the peripharyngeal glands. Morphological drawings were then performed under a compound light microscope for illustration.

### Molecular analysis

Two trematodes were individually placed into 1.5-ml microcentrifuge tubes and washed thoroughly with sterile distilled water. Total genomic DNA was isolated using the Geneaid gDNA DNA Mini Kit (Geneaid Biotech Ltd, Taipei, Taiwan) following the manufacturer’s recommendations.

Polymerase chain reaction amplifications of the partial nuclear 28S rRNA gene, partial mitochondrial cytochrome *c* oxidase subunit 1 (*cox1*) gene, and the complete nuclear internal transcribed spacer 2 (ITS2) region were performed in a final reaction volume of 30 µl. Each reaction contained 15 µl of 2x i-Taq™ mastermix (iNtRON Biotechnology, Gyeonggi, South Korea), 10 to 50 µm of each primer, and 1µl (1ng) of DNA template. PCR was conducted in a T100™ thermocycler (Bio-Rad, California, USA), and the thermocycling conditions followed the respective publications of the primers. The primer sequences are: 28S rRNA (Digl2: 5’ – AAGCATATCACTAAGCGG – 3′, 1500R: 5′ – GCTATCCTGAGGGAAACTTCG – 3′) (Tkach et al. 2003), *cox1* (JB3: 5′ – TTTTTTGGGCATCCTGAGGTTTAT – 3′, JB4.5: 5′ – TAAAGAAAGAACATAATGAAAATG – 3′) (Bowles et al. 1992) and ITS2 (5.8SF: 5’ – CTCGTGTGTCGATGAAGAG – 3’, 28S4R: 5’ – TATTTAGCCTTGGATGGAGTTTACC – 3’) (Besprozvannykh et al. 2019). PCR amplicons were visualized on 1% agarose gel stained with SYBR™ Safe DNA Gel Stain (Invitrogen, Massachusetts, USA). The amplicons were sent for Fast Next Generation Sequencing with a commercial company (Tsingke Biotech, Beijing, China).

The electropherograms obtained after sequencing were checked with BioEdit 7.0 (Hall 1999), and multiple sequence alignment for each genetic marker was performed using ClustalX 2.1 (Thompson et al. 2002) with reference sequences in family Plagiorchiidae obtained from the NCBI database. *Tremiorchis ranarum* and *Orientocreadium* sp. were used as the outgroup taxa. For the concatenated sequences, the sequence per sample from each genetic marker was manually combined into one sequence, followed by multiple sequence alignment. Phylogenetic analyses with the maximum likelihood (ML) and Bayesian Inference (BI) methods were performed in MEGA X (Kumar et al. 2018) and MrBayes 3.2, respectively (Huelsenbeck and Ronquist 2001), respectively. For ML, the best-fit nucleotide substitution model with 1000 bootstrap iterations was used. BI was conducted using four MCMC chains for 1,000,000 generations and a sampling frequency of every 100 generations using the same best-fit nucleotide substitution model as the ML phylogenies. Bayesian posterior probability values were calculated after discarding the initial 25% of trees as ‘burn-in’. The phylogenies were visualized and labeled using FigTree 1.3.1 (Rambaut 2009). Interspecies genetic distances using the pairwise distance model were calculated in MEGA X (Kumar et al. 2018).

## Results

### Morphological identification

Of the three *H. rugulosus* examined, two individuals were found to be infected with *G. staffordi*, with a prevalence of 66.7% and mean intensity of 12 trematodes per host. The trematodes were found in the mesentery and intestine. Morphological identification confirmed that the specimens obtained are *G. staffordi*, based on the presence of a Y-shaped excretory vesicle, absence of peripharyngeal glands, ovary situated at the level of the ventral sucker, and having follicular vitelline follicles that begin at the level of the ventral sucker to 3/4 of the body. Additionally, *G. staffordi* is the only species recorded from amphibian hosts in Southeast Asia. The morphological measurements of *G. staffordi* obtained in this study are summarized in Table 1 and Supplementary file 1.

**Table 1:** Morphological comparisons of *G. staffordi* (Tubangi, 1928) with the specimens from this study

Reference	This study	Cheng 1959	Yuen 1962	Fischthal and Kuntz 1967
Location	Thailand (Bangkok)	The Philippines (Luzon)	Malaysia, Singapore	The Philippines (Luzon, Manila)
Host	*Hoplobatrachus rugulosus*	*Rana vittigera*	*Rana macrodon*,* Rana erytharea*,* Rana cancrivora*	*Rana vittigera*
Site of infection	Mesentery, intestine	Intestine	Intestine	Stomach, intestine
No. of specimens	15	NA	9	8
Body length	2150–4133 (2780)	2090–4150	1940–4960	1454–1960
Body width	883–1207 (1053)	630–1010	610–1570	397–625
Body width: body length ^a^	3:1	3:1	3:1	3:1
Oral sucker	200–340 (256) ⋅ 183–292 (237)	190–290	190–360	151–206 ⋅ 155–222
Ventral sucker	91–203 (155) ⋅ 73–182 (123)	150–180	130–250	80–125 ⋅ 96–130
Oral sucker: ventral sucker ^a^	1:0.6	1:0.6–0.7	1:0.6	1:0.48–0.67
Pharynx	108–216 (152) ⋅ 103–215 (147)	NA	110–170	75–111 ⋅ 75–121
Anterior testis	233–499 (348) ⋅ 161–356 (219)	180–220	230–380 ⋅ 130–380	157–206 ⋅ 160–206
Posterior testis	239–421 (319) ⋅ 176–355 (247)	180–220	230–380 ⋅ 130–380	162–215 ⋅ 114–189
Ovary	187–458 (301) ⋅ 143–295 (203)	130–180	130–420 ⋅ 190–390	111–143 ⋅ 114–186
Cirrus sac	146–277 (216) ⋅ 93–159 (123)	260–330 × 60–90	NA	99–182 ⋅ 63–87
Vitelline follicles	Follicular, ventral sucker to 3/4 of body	Follicular, ventral sucker to 3/4 of body	Follicular	NA
Uterus	Intercaecal, some overlap with caeca	NA	Intercaecal, so overlap with caeca	NA
Egg	22–28 (24) ⋅ 12–18 (15)	31–33 ⋅ 15–18	21–33 ⋅ 17–22	29–37 ⋅ 19–27
Excretory vesicle	Y-shaped	Y-shaped	Y-shaped	NA

^a^The ratios were calculated based on the minimum and maximum values for body length, body width, oral sucker, and ventral sucker. NA indicates no information was available. All measurements are in µm, the average is in parentheses

Comparison of the morphological measurements of *G. staffordi* from other studies (Table 1) revealed an additional host habitat of *G. staffordi* aside from the intestine and stomach. *Glypthelmins staffordi* was also found in the host’s mesentery. Variation in the size of testis and ovary was also observed, where the maximum diameter of the specimens obtained was larger than those from previous studies. However, other morphological measurements (e.g. the ratio of body width to body length, ratio of oral sucker to ventral sucker, and egg size) were consistent. These measurements, alongside other morphological characteristics of *G. staffordi* show congruence between studies.

**Fig. 1:**
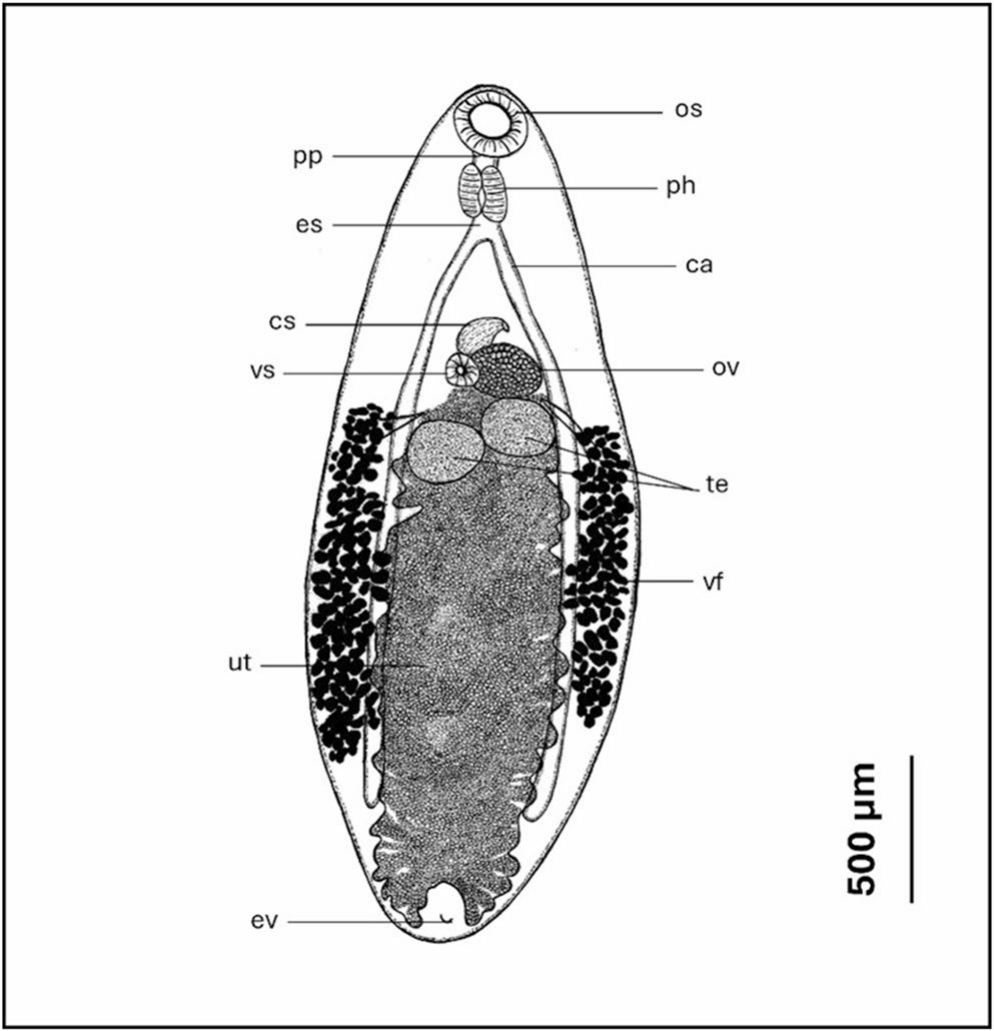
Morphological drawing of *Glypthelmins staffordi* obtained from *Hoplobatrachus rugulosus* in Thailand. Abbreviations: os, oral sucker; pp, pre-pharynx; ph, pharynx; es, esophagus; ca., intestinal caeca; cs, cirrus sac; vs., ventral sucker; ov, ovary; te, testes; vt, vitelline follicles; ut, uterus; ev, excretory vesicle

Based on 15 specimens (measurements presented as range of the values described in µm, followed by the mean in parentheses), the illustration is presented in Fig. 1 and redescribed as follows: Body oval, elongate, 2150–4133 (2780) long, maximum width 883–1207 (2780), 3:1 width to length ratio. Body tegument spinous. Oral sucker subterminal; 200–340 (256) ⋅ 183–292 (237), larger than ventral sucker. Ventral sucker small, positioned in anterior third of body next to ovary; 91–203 (155) ⋅ 73–182 (123). Ratio of oral sucker to ventral sucker 1:0.6. Pre-pharynx and esophagus short, pharynx measures 108–216 (152) ⋅ 103–215 (147). Peripharyngeal glands absent. Intestinal caeca extend to posterior 1/5 of body. Testes round to transversely elongate, anterior testis positioned directly below ovary. Anterior testis measures 233–499 (348) ⋅ 161–356 (219), posterior testis measures 239–421 (319) ⋅ 176–355 (247). Cirrus sac large, curved, situated above ventral sucker, and measures 146–277 (216) ⋅ 93–159 (123). Vitelline follicles follicular, extends from level of ventral sucker to 3/4 of body. Uterus fills most of the intracaecal space after the level of the testes to the posterior of body and is filled with eggs. Eggs 22–28 (24) ⋅ 12–18 (15). Excretory vesicle Y-shaped.

### Taxonomic summary

- **Host**: *Hoplobatrachus rugulosus* Wiegmann, 1834 (Anura: Dicroglossidae)
- **Locality:** Min Buri, Bangkok, Thailand
- **Site of infection:** Mesentery and intestine
- **Prevalence and mean intensity:** 66.7% (2 out of 3 hosts infected), 12 trematodes per host
- **Specimens deposited:** Department of Helminthology, Faculty of Tropical Medicine, Mahidol University (Identification no. 2025-TR-Glypthelmins-1 to 2025-TR-Glypthelmins-15)

With the inclusion of *G. staffordi*, the genus now contains 12 species, including *G. eleutherodactyli*, which was newly described by Moravec and Prouza (2024) from *Eleutherodactylus* sp. in Venezuela (Moravec and Prouza 2024). Table 2 presents the morphological comparison of eight other *Glypthelmins* species (those without peripharyngeal glands), and an updated key to the 12 recognized species of *Glypthelmins* Stafford 1905 is provided below:

- 1a – Peripharyngeal glands present ------------------------------------------- 2.
- 1b – Peripharyngeal glands absent -------------------------------------------- 4.
- 2a – Vitelline follicles scarce, distributed extracaecally and never converging --------------------------------------------------------G. pennsylvaniensis
- 2b – Vitelline follicles not scarce, often overlapping with intestinal caeca --------------------------------------------------------------------------- 3.
- 3a –Peripharyngeal glands large and prominent, often extending from anterior border of pharynx to slightly beyond intestinal caeca bifurcation ------------------------------------------------G. quieta.
- 3b – Peripharyngeal glands smaller, not extending beyond intestinal bifurcation --------------------------------------------------------G. hyloreus.
- 4a – Body width to length ratio approximately 10:1; testes positioned in tandem --------------------------------------------------------------------G. intestinalis.
- 4b – Body width to length ratio approximately 4:1; testes positioned diagonally –------------------------------------------------------ 5.
- 5a – Excretory vesicle Y-shaped -------------------------------------------------G. staffordi.
- 5b – Excretory vesicle I-shaped or indistinct ---------------------------------- 6.
- 6a – Cirrus sac straight, not curved in shape ----------------------------------G. shastai.
- 6b – Cirrus sac curved in shape -------------------------------------------------- 7.
- 7a – Vitelline follicles begin from level of oral sucker to posterior testis ----------------------------------------------------------------G. californiensis.
- 7b – Vitelline follicles begin from level of intestinal bifurcation or cirrus sac –------------------------------------------------------------------ 8.
- 8a – Oral sucker slightly larger than ventral sucker (ratio 1:0.7–0.9) ---------G. parva.
- 8b – Oral sucker significantly larger than ventral sucker (ratio 1:0.4–0.6) -------------------------------------------------------------- 9.
- 9a – Vitelline follicles extend beyond the level of posterior testis ---------------- 10.
- 9b – Vitelline follicles terminate at the level of posterior testis -------------------- 11.
- 10a – Uterus at the posterior end extends beyond inner margin of intestinal caeca -------------------------------------------------G. tuxtlasensis.
- 10b– Uterus at posterior end does not reach to the inner margin of intestinal caeca -------------------------------------------------G. eleutherodactyli.
- 11a– Uterus at the posterior end extends beyond inner margin of intestinal caeca -------------------------------------------------------------G. facioi.
- 11b– Uterus at posterior end does not reach to inner margin of intestinal caeca -------------------------------------------------------------G. brownorumae.

**Table 2:** Morphological measurements for the eight other *Glypthelmins* species

Reference	*G. shastai* (Ingles 1936)	*G. parva* (Travassos 1924)	*G. eleutherodactyli* (Moravec and Prouza 2024)	*G. tuxtlasensis* (Razo-Mendivil et al. 2004)	*G. brownorumae* (Razo-Mendivil et al. 2004)	*G. facioi* (Brenes et al. 1959)	*G. californiensis* (Cort 1919)	*G. intestinalis* (Lucker 1931) O’’Grady, 1987
	Ingles 1936	Cheng 1959	Moravec and Prouza 2024	Razo-Mendivil et al. 2004	Razo-Mendivil et al. 2004	Sullivan 1976	Cheng 1959	Lucker 1931
Location	United States of America (California)	Brazil	Venezuela	Mexico (Veracruz)	Mexico (Tabasco)	Costa Rica (Cartago)	United States of America (California)	United States of America (Washington)
Host	*Bufo boreas*	*Cystignathus ocellatus*	*Eleutherodactylus* sp.	*Rana vaillanti*	*Rana brownorum*	*Rana pipiens*	*Rana aurora*	*Rana pretiosa*
Site of infection	Intestine	Intestine	Digestive tract	Anterior intestine	Anterior intestine	Small intestine	Small intestine	Intestine
No. of specimens	NA	NA	10	18	15	29	NA	10
Body length	1200–2300	(1300)	4080–7090	1440–2770	1500–2125	1820–3440 (2800)	2400–5000	5000
Body width	540–770	(460)	1400–1710	314–614	328–528	390–900 (640)	640	480–630
Body width: body length ^a^	2:1	2:1	3:1	4:1	4:1	4:1	3:1	10:1
Oral sucker	130–220	(140)	408–490 ⋅ 455–571	158–252 ⋅ 162–270	148–209 ⋅ 169–216	170–240 (200) ⋅ 170–250 (220)	400	256–320
Ventral sucker	120–160	(130)	163–286 ⋅ 163–286	86–144	92–119 ⋅ 97–129	90–140 (120) ⋅ 80–150 (120)	240–310 ⋅ 250–410	176–240
Oral sucker: ventral sucker ^a^	1:0.7–0.9	1:0.9	1:0.4–0.58	1:0.48–0.65	1:0.55–0.67	1:0.58	1:0.6	1:0.6–0.7
Pharynx	100–130	NA	286–326 ⋅ 340–442	79–133 ⋅ 104–158	97–122 ⋅ 72–119	70–110 (90) ⋅ 110–160 (130)	NA	110–140 ⋅ 80–100
Anterior testis	220–290	150–170	272–394 ⋅ 354–517	90–234 ⋅ 79–199	137–198 ⋅ 104–148	130–240 (200) ⋅ 100–230 (170)	NA	320–368
Posterior testis	220–290	150–170	313–503 ⋅ 354–476	83–238 ⋅ 72–228	144–212 ⋅ 104–158	130–290 (240) ⋅ 110–250 (190)	NA	320–368
Ovary	130–190	NA	313–340 ⋅ 272–367	83–228 ⋅ 68–221	104–137 ⋅ 75–119	100–240 (170) ⋅ 90–210 (160)	NA	220–240
Cirrus sac	260–400 ⋅ 80–110	NA	326–517 ⋅ 231–286	162–399 ⋅ 54–119	108–234 ⋅ 68–97	150–340 (270) ⋅ 50–100 (70)	NA	NA
Vitelline follicles	Follicular, genital pore to 2/3 of body	Ventral sucker to posterior testes	Cirrus sac to mid length between posterior testis to body end	Follicular, intestinal bifurcation to after posterior testis	Follicular, intestinal bifurcation to posterior testis	Follicular, intestinal bifurcation to posterior testis	Follicular, oral sucker to posterior testis	Follicular, ovary to 4/5 of body
Uterus	Intercaecal	NA	Intercaecal, does not reach beyond inner margin	Intercaecal, some loops reach beyond inner margin	Intercaecal, does not reach beyond inner margin	Intercaecal, extend beyond inner margin	NA	Intercaecal, some overlap with caeca
Egg	31–32 ⋅ 14–17	26–38 ⋅ 11–18	27–30 ⋅ 12–15	21–28 ⋅ 14–18	32–39 ⋅ 14–21	27–32 (29) ⋅ 12–20 (15)	46–56 ⋅ 19–26	48–58 ⋅ 18–26
Excretory vesicle	I-shaped	I-shaped	Indistinct	I-shaped	I-shaped	I-shaped	I-shaped	I-shaped

^a^The ratios were calculated based on the minimum and maximum values for body length, body width, oral sucker, and ventral sucker. NA indicates no information was available. All measurements are in µm, the average is in parentheses

### Molecular validation

**Fig. 2:**
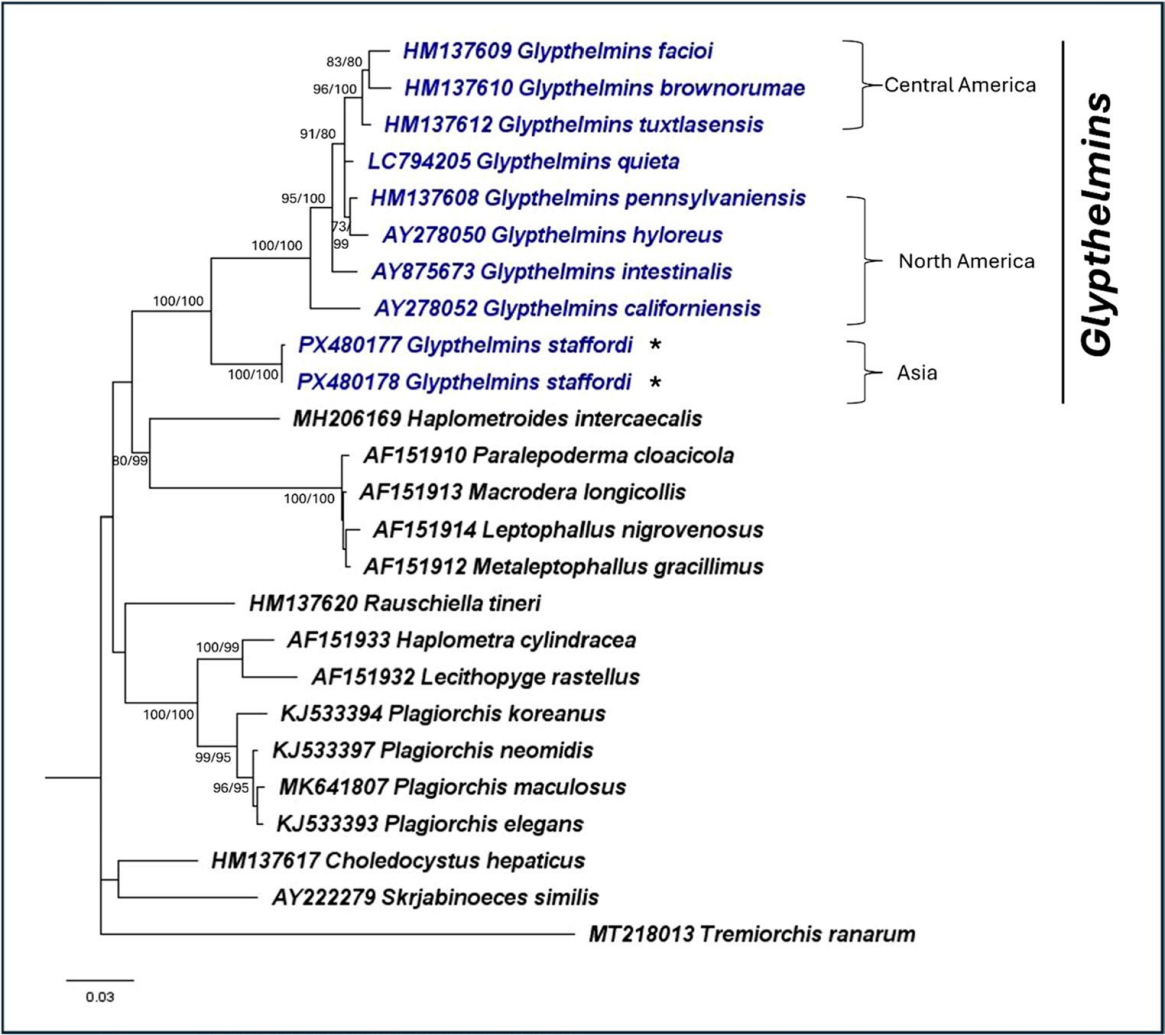
Maximum likelihood phylogeny of Plagiorchiidae using the nuclear 28S rRNA gene (GTR + G). A total of 24 ingroup and 1 outgroup taxa was included in the analysis. There was a total of 979 nucleotide positions for each sequence. Numbers at nodes indicate posterior probability/bootstrap values of BI/ML, where values of more than 70 are indicated. Species in *Glypthelmins* are in blue, while the representative *G. staffordi* specimens are indicated with an “*”. Species in the three geographical clades are indicated by the square brackets

**Fig. 3:**
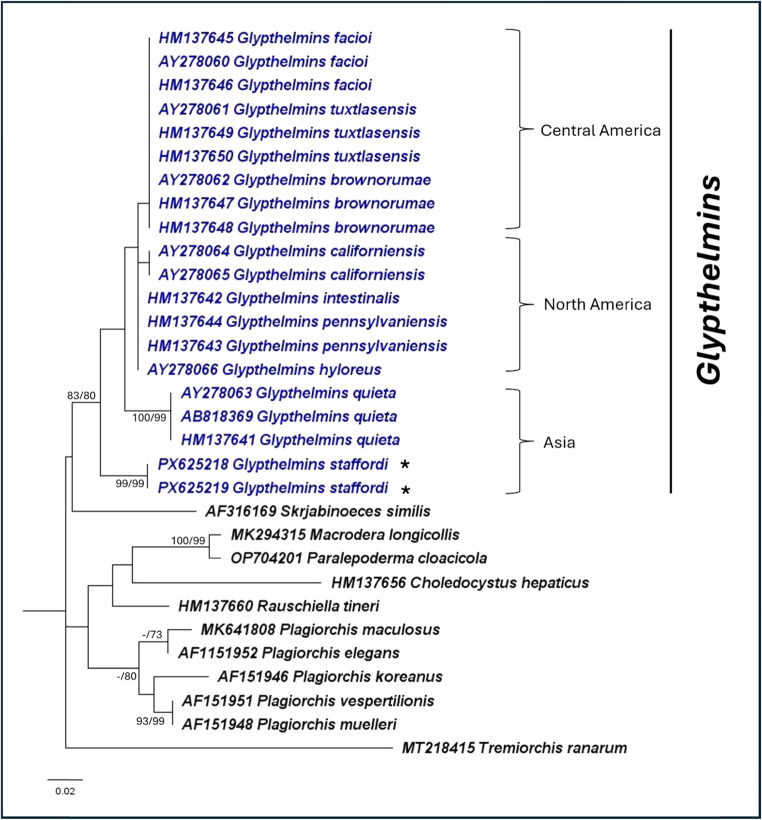
Maximum likelihood phylogeny of Plagiorchiidae using the nuclear ITS2 region (K2 + I). A total of 30 ingroup and 1 outgroup taxa was included in the analysis. There was a total of 156 nucleotide positions for each sequence. Numbers at nodes indicate posterior probability/bootstrap values of BI/ML, where values of more than 70 are indicated. Species in *Glypthelmins* are in blue, while the representative *G. staffordi* specimens are indicated with an “*”. Species in the three geographical clades are indicated by the square brackets

**Fig. 4:**
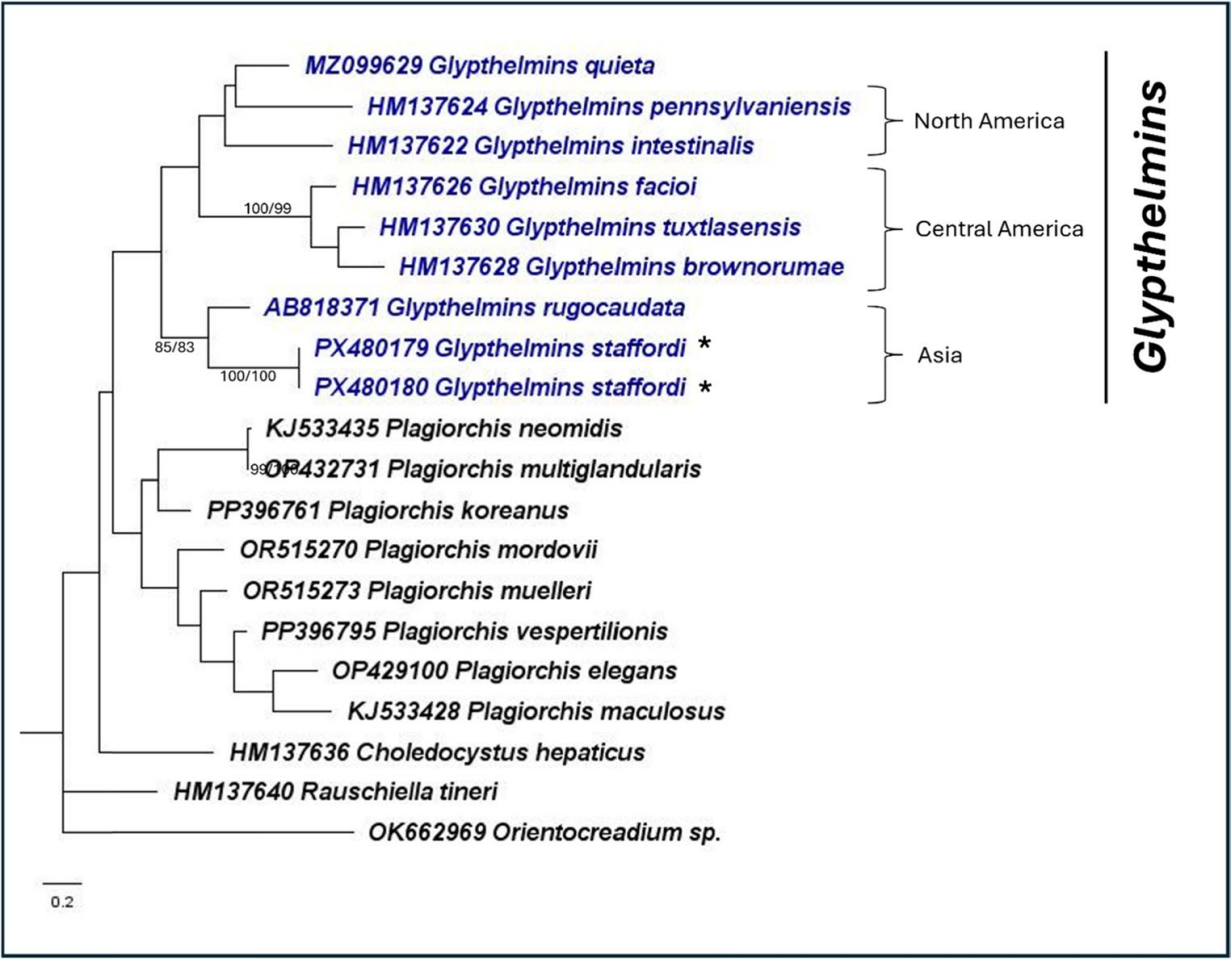
Maximum likelihood phylogeny of Plagiorchiidae using the mitochondrial *cox1* gene (TN + G). A total of 19 ingroup and 1 outgroup taxa was included in the analysis. There was a total of 339 nucleotide positions for each sequence. Numbers at nodes indicate posterior probability/bootstrap values of BI/ML, where values of more than 70 are indicated. Species in *Glypthelmins* are in blue, while the representative *G. staffordi* specimens are indicated with an “*”. Species in the three geographical clades are indicated by the square brackets

**Fig. 5:**
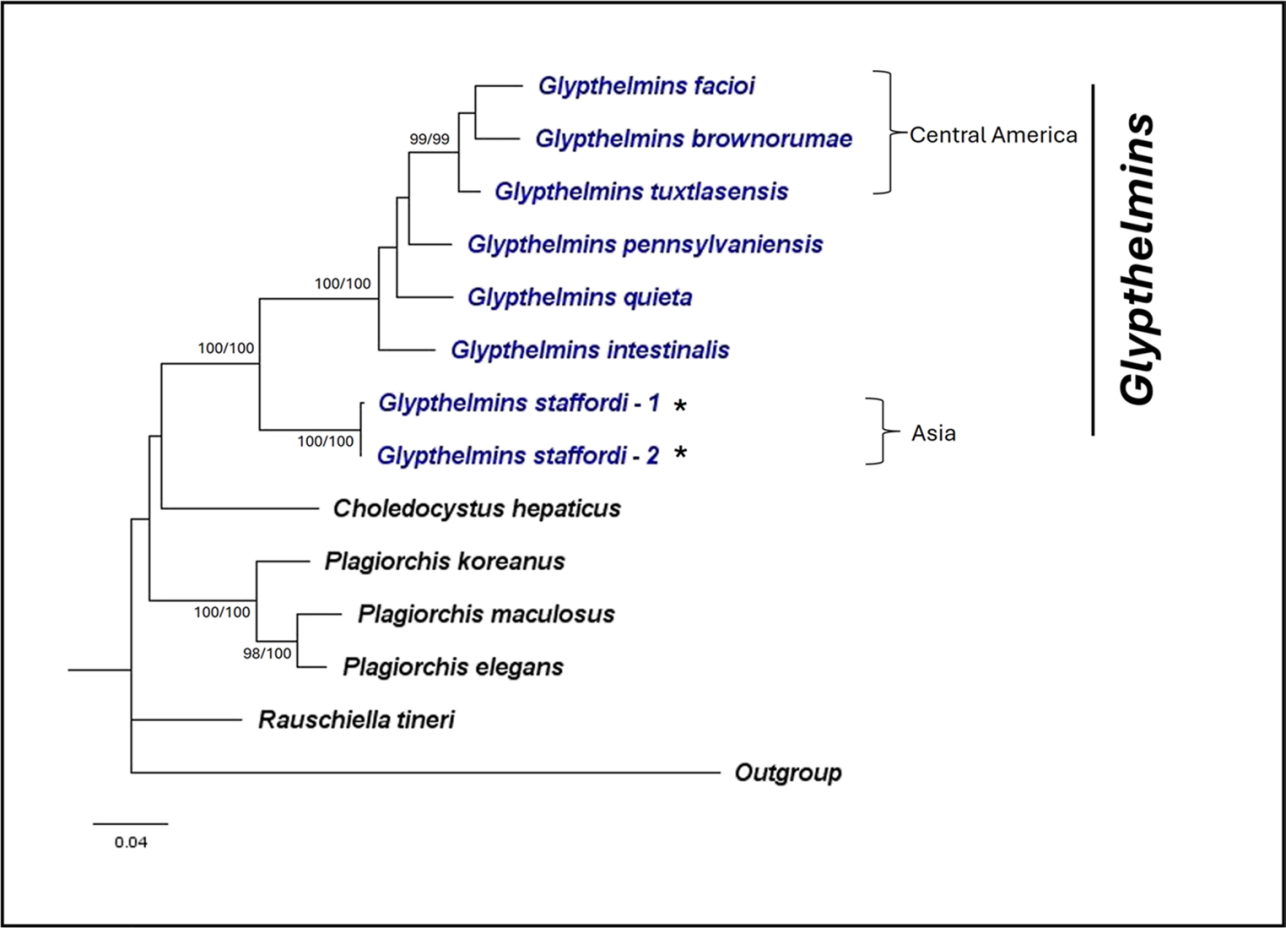
Maximum likelihood phylogeny of Plagiorchiidae using the concatenated sequences of the nuclear 28S, ITS2, and mitochondrial **cox1** markers (GTR + G). A total of 13 ingroup and 1 outgroup taxa was included in the analysis. There was a total of 1474 nucleotide positions for each sequence. Numbers at nodes indicate posterior probability/bootstrap values of BI/ML, where values of more than 70 are indicated. Species in *Glypthelmins* are in blue, while the representative *G. staffordi* specimens are indicated with an “*”. Species in the three geographical clades are indicated by the square brackets

To confirm that the specimens obtained are *G. staffordi*, three genetic markers (28S rRNA, ITS2, and *cox1*) were used. Figures 2, 3 and 4 presents the ML phylogenies obtained, while the sequences used for analyses are in Supplementary file 2. Across all three phylogenies, *Glypthelmins* was monophyletic, including *G. staffordi*. Also, *G. staffordi* was the most genetically different *Glypthelmins* as compared to the other included species, providing evidence that the *Glypthelmins* specimens in this study are a different species from the reference sequences. A similar phylogeny was also observed using the concatenated sequences of all three genetic markers (Fig. 5). The genetic distances obtained between *G. staffordi* and other *Glypthelmins* species were 7.3–8.2%, 3.8–5.1%, and 12.3–18.8%, for the 28 S, ITS2, and *cox1* markers, respectively. The genetic distances obtained for the genetic markers are in Supplementary file 3. Additionally, the phylogenies support that *G. staffordi* does not belong to the genus *Rauschiella.*

Within *Glypthelmins*, the phylogenies support the distinction of species based on their geographic distribution. For example, *G. facioi*, *G. brownorumae*, and *G. tuxtlasensis*, which infect Central America anurans form a clade in all three phylogenies. The species (*G. pennsylvaniensis*, *G. californiensis*, *G. hyloreus*, and *G. intestinalis*) which occur in frogs from North America are genetically closer together. *Glypthelmins staffordi*, which have been found in frogs from Southeast Asia are a sister group to other species, demonstrating genetic distinction within the genus based on geographic localities.

Within Plagiorchiidae, *Glypthelmins* is sister to *Haplometroides*, *Paralepoderma*, *Macrodera*, *Leptophallus*, and *Metaleptophallus* in the 28S rRNA gene phylogeny. Genetic distances ranged between 11.5% – 14.1%, with *Haplometroides* genetically closest to *Glypthelmins*. *Rauschiella*, *Haplometra*, *Lecithopyge*, and *Plagiorchis* formed another clade, while *Choledocystus* and *Skrjabinoeces* form a sister clade to all other genera in the phylogeny. The ITS2 region phylogeny showed a different relationship, where *Glypthelmins* is sister to *Skrjabinoeces*, while *Macrodera*, *Paralepoderma*, *Choledocystus*, *Rauschiella*, and *Plagiorchis* all form another clade. Similarly, a different relationship was observed using the *cox1* gene phylogeny, where *Choledocystus* and *Rauschiella* were the most distantly related genera to *Glypthelmins.*

## Discussion

Through morphological comparisons and molecular analysis, *G. staffordi* was identified infecting *H. rugulosus* from Thailand. Although *G. staffordi* has been previously described and reported in the same host, those descriptions date back several decades and lack molecular information. Moreover, the current taxonomic status of *G. staffordi* is complicated, and is not considered a valid species in *Glypthelmins*. Here, our results provide the first molecular data for *G. staffordi* and support its recognition as a valid species in genus.

Based on Razo-Mendivil et al. (2006), *G.* *staffordi* was assigned to *Rauschiella* based on morphological characters (e.g. the Y-shaped excretory vesicle) (Razo-Mendivil et al. 2006). Here, the nuclear and mitochondrial phylogenies obtained in this study revealed that *G. staffordi* is genetically closer to other species in *Glypthelmins* than to *Rauschiella*. *Glypthelmins staffordi* formed a well-supported monophyletic clade alongside other *Glypthelmins* species, further supporting placement of *G. staffordi* in the genus *Glypthelmins*. Additionally, the genetic distance values also provide support, with larger genetic distances obtained between *G. staffordi* and *Rauschiella tineri*, as compared between *G. staffordi* and other *Glypthelmins*. This is similar to *G. intestinalis*, which was previously placed in *Haplometrana* before being transferred to *Glypthelmins* based on morphological, allozyme, and molecular analyses (Brooks and McLennan 1993; Razo-Mendivil et al. 2006). The use of morphology alone may not be sufficient for elucidating systematic relationships within this genus, thus advocating the importance of integrative taxonomy for *Glypthelmins*. Incorporating integrative taxonomy and the use of various genetic markers may also aid in shedding light on evolutionary relationships within Plagiorchiidae, where varied relationships between genera were observed from the phylogenies obtained from this study. Moreover, the shape of the excretory vesicle appears unreliable as a diagnostic morphological character distinguishing between genera.

Morphological identification of *Glypthelmins* relies on characters such as the shape of the excretory vesicle, uterus position, uterine loops, and the position of vitelline follicles. However, intra species morphological variations may exist (Razo-Mendivil and Pérez-Ponce de León 2008). Morphological variations render the differentiation of closely related species challenging especially if their geographical distribution overlaps. For example, *G. brownorumae* and *G. tuxtlasensis*, which were both described in Mexico from *Rana brownorum* and *R. vaillanti*, respectively, exhibit minor morphological differences (Razo-Mendivil et al. 2004). They differ in the width of the esophagus, egg size, position of vitelline follicles, and position of uterine loops (Razo-Mendivil et al. 2004). These morphological differences may not be apparent and may be subjective depending on the mounting procedures. Additionally, morphological homoplasy, where similar morphological characters develop due to shared ecological conditions may complicate its use (Bray et al. 2016).

Integrating molecular data has therefore been crucial to shed light on this taxonomically challenging genus. Previous studies provided nuclear and mitochondrial sequences of the valid species in *Glypthelmins*, supporting its monophyly (Razo-Mendivil et al. 2004, 2006; Razo-Mendivil and Pérez-Ponce de León 2011). Additionally, unusual levels of morphological homoplasy were suggested, which may be a result of host sharing and leading to host induced variation (Razo-Mendivil and Pérez-Ponce de León 2011). Molecular information also suggested a distinct biogeographic structure within *Glypthelmins*, separating species from the Nearctic and Neotropical zones. Similarly, the present phylogenetic analyses obtained support this distinction, with the North American species and Central American species clustered separately. Moreover, *G. staffordi*, which have been found exclusively in Asia, was recovered as a sister lineage to both groups, indicative of geographical divergence within the genus.

Despite the advantages of molecular information, appropriate genetic markers should be selected, with markers selected from different loci (Chan et al. 2024; Thaenkham et al. 2022). Among the three genetic markers used, highest sequence variation between species was found using the mitochondrial *cox1* gene, as compared to the nuclear markers. The results are congruent with the findings from Razo-Mendivil et al. (2011), with interspecies genetic distances ranging from 10.2 to 19.7% using the *cox1* gene (Razo-Mendivil et al. 2011). Moreover, no sequence variation was observed in the ITS2 sequences between the morphologically similar *G. brownorumae*, *G. tuxtlasensis*, and *G. facioi*, where these three species have been previously described from frogs in Central America. These results suggest potential cryptic diversity, where mitochondrial genetic markers (e.g. *cox1*) may provide greater resolution for species delimitation.

*Hoplobatrachus rugulosus* is widely distributed across Thailand, commonly inhabiting wetlands, marshes, ponds, and rice paddy fields (Traijitt et al. 2021). Its distribution extends to other Southeast Asian countries, including Myanmar, Lao People’s Democratic Republic, Vietnam, Cambodia, and Malaysia (Frost 2025). In Thailand, *H. rugulosus* is economically, ecologically, and scientifically important (Madhyamapurush et al. 2024; Traijitt et al. 2021). Aside from *G. staffordi*, several parasites have been recorded infecting *H. rugulosus*, including blood protozoans such as *Trypanosoma* and *Hepatozoon* (Sailasuta et al. 2011). Infection can result in inflammatory lesions despite the absence of clinical symptoms. Similarly, although infection with *Glypthelmins* has not been reported to cause any visible clinical symptoms in frogs, heavy infections may potentially influence the frog’s development and survival. Additionally, human parasitic helminths such as *Gnathostoma* and *Spirometra* have been reported infecting *H. rugulosus* in Myanmar and Cambodia, highlighting the zoonotic risk associated with the consumption of undercooked frogs (Chai et al. 2020; Jongthawin et al. 2014; Sohn et al. 2021).

## Conclusion

 Through molecular validation using nuclear and mitochondrial genetic markers, alongside morphological comparisons, *G. staffordi* is proposed to be a valid species in *Glypthelmins*. This study provides the first molecular data for *G. staffordi* infecting *H. rugulosus* in Thailand, affirming its position as a sister clade to the Neotropical and Neoarctic species. Future surveys in amphibians and the use of molecular information are advocated, which will allow a better understanding of the taxonomy and evolutionary history of this group of taxonomically complex digeneans

## Supplementary Information

Below is the link to the electronic supplementary material.

Supplementary Material 1 Morphological measurements of *Glypthelmins staffordi *(XLSX 17.7 KB)

Supplementary Material 2 Details of sequences used for phylogenetic analyses (XLSX 16.0 KB)

Supplementary Material 3 Inter and intra species genetic distances obtained using p-distance (DOCX 14.7 KB)

## Data Availability

All data generated in this study are included in the published article and its supplementary files. The sequences have been deposited in the NCBI database under the accession numbers PX480177 and PX480178 for the 28S rRNA gene, PX480178 and PX480179 for the *cox1* gene, and PX625218 and PX625219 for the ITS2 region.
