# Supplementary material for: Molecular validation and redescription of Glypthelmins staffordi (Digenea: Plagiorchiidae) with an updated key to genus Glypthelmins Stafford, 1905
Source: Parasitol Res. 2026 Apr 10;125(1):58. doi: 10.1007/s00436-026-08668-4 (PMC13171979; doi:10.1007/s00436-026-08668-4)
Supplement: Supplementary file 3 — Supplementary Material 3 Inter and intra species genetic distances obtained using p-distance (DOCX 14.7 KB) [file 436_2026_8668_MOESM3_ESM.docx]

**Supplementary file 3. Inter and intra species genetic distances obtained using *p*-distance**

| Comparison | Genetic marker | | | |
| --- | --- | --- | --- | --- |
|  | 28S | ITS2 | *COI* | concatenated |
| Within *G. staffordi* | 0.1 | 0 | 0.2 | 0.1 |
| Between *G. staffordi* and other *Glypthelmins* | 7.3 – 8.2 (7.6) | 3.8 – 5.1 (4.3) | 12.3 – 18.8 (16.5) | 8.7 – 9.7 (9.4) |
| Between *G. staffordi* and *R. tineri* | 9.0 – 9.1 (9.1) | 8.9 | 17.1 – 17.4 (17.3) | 10.9 – 11.0 (10.9) |

Values are presented as percentages, with the average in parentheses.
